# Sleep disturbances in anorexia nervosa

**DOI:** 10.1002/erv.3148

**Published:** 2024-10-23

**Authors:** Pia Burger, Rosita W. Bos, Joyce Maas, Mladena Simeunovic‐Ostojic, Reinoud J. B. J. Gemke

**Affiliations:** ^1^ Center for Eating Disorders Helmond Mental Health Center Region Oost‐Brabant Helmond The Netherlands; ^2^ Department of Pediatrics Emma Children's Hospital Amsterdam UMC Amsterdam Netherlands; ^3^ Amsterdam Public Health Research Institute Amsterdam UMC Vrije Universiteit Amsterdam Amsterdam The Netherlands

**Keywords:** anorexia nervosa, co‐morbidity

## Abstract

**Introduction:**

Sleep is crucial for physical and psychological health, and disturbances are closely linked to psychiatric disorders, making their management essential for improving treatment outcomes and preventing relapse. Although sleep disturbances are implicated in psychopathology of eating disorders, its role in anorexia nervosa (AN) remains unclear. This review aimed to characterise sleep in AN.

**Methods:**

A systematic search was conducted in four scientific databases, including papers from inception to 25 May 2024. A machine learning algorithm (ASReview) was utilised to screen titles and abstracts for eligibility. Sleep quantity, architecture, and quality were investigated. Meta‐analyses were conducted to investigate the difference between patients with AN and healthy controls (HC) in total sleep time (TST), wake after sleep onset (WASO), sleep onset latency (SOL), sleep efficiency, Sleep Stage 1, 2, slow wave sleep, rapid eye movement (REM) sleep and REM latency. Certainty of evidence was assessed using the GRADE approach.

**Results:**

Out of 67 potentially eligible papers, 31 were included in this review, with 15 in the meta‐analyses. Statistically significant average mean differences were found for TST (−32.1 min [95% CI: −50.9, −13.4]), WASO (19.0 min [95% CI: −2.4, 40.3]), and sleep efficiency (−4.4% [95% CI: −7.9, −0.9]). Additionally, Sleep stage 1 was significantly increased (2.4% [95%‐CI: 0.05, 4.7]), while REM sleep was reduced (−2.1% [95%‐CI: −4.2, −0.02]). Subgroup analysis showed that TST and WASO did not improve significantly after weight restoration.

**Conclusion:**

Sleep in patients with AN is impaired, with lower TST and sleep efficiency, higher WASO, more time in stage 1 sleep, and less in REM. Weight restoration alone may not improve sleep. While more research is needed, substantial accompanying sleep disturbances in AN justifies addressing these in current treatment practice, also because of the chronic character of AN and importance of sleep for long term (mental) health.

## Highlights

- Patients with anorexia nervosa experience longer periods of wakefulness during the night, along with lower total sleep time and sleep efficiency compared to healthy controls.
- Patients with anorexia nervosa spend more time in stage 1 sleep and less time in rapid eye movement sleep compared to healthy controls.
- Weight restoration alone is not sufficient to improve sleep.

## 1 INTRODUCTION

Sleep is an essential daily requirement for the development and maintenance of physical health and psychological functioning (Alvarez & T, 2004). Among the important functions of sleep are memory processing and consolidation, cellular repair, brain development and hormonal regulation (Dinges, 2006; Kopasz et al., 2010; Leproult & Van Couter, 2010; Stickgold & Walker, 2007; Tononi & Cirelli, 2006). Consequences of impaired sleep include reduced alertness, poor emotion regulation, difficulties with decision making, and anxiety (Alvarez & T, 2004; Chorney et al., 2008; Medic et al., 2017). Examples of sleep disturbances include insufficient and fragmented sleep, excessive sleep, shifts in sleep timing, and parasomnias.

Sleep disturbances are suggested to be a transdiagnostic phenomena that have an etiological link to several psychiatric disorders (e.g., depression, bipolar disorder, psychotic and anxiety disorder) through different pathways (Harvey et al., 2011), and with evidence suggesting a bidirectional association (Abad & Guilleminault, 2005; Freeman et al., 2020; Ivanenko & Johnson, 2008). The associations between sleep disturbances and psychiatric disorders are likely to reflect an overlap in genetic, neurobiological, psychological, and environmental causes (Gregory et al., 2016; Lind et al., 2017; Riemann et al., 2020; Sinha, 2016; Wulff et al., 2010).

Sleep plays an important role in the regulation of body weight, with the amount of sleep and synchronization of the biological clock being necessary for achieving energy balance and hormone secretion related to weight regulation (Leger et al., 2015/12). Additionally, food intake is an essential element in regulating sleep (Binks et al., 2020). Starvation or malnutrition can lead to sleep disturbances due to increased levels of the hormone orexin. For example, orexin is one of the key regulators of arousal and wakefulness, affecting autonomic functions (e.g., increased heart rate and blood pressure) and metabolic processes (e.g., metabolic rate and glucose metabolism). It also influences reward and motivation behaviour related to food intake (Allison et al., 2016; Hagen et al., 2015; Janas‐Kozik et al., 2011; Tauman, 2012). Sleep is also involved in modulating the synthesis and concentration of ghrelin and leptin, two hormones that regulate appetite and energy balance. Studies have shown that inadequate sleep can significantly affect these hormones, by increasing ghrelin levels (which stimulate appetite), and decreased leptin levels (which suppresses appetite) (Van Cauter & Knutson, 2008). Elevated ghrelin levels are associated with increased wakefulness and reduced sleep‐wave sleep (SWS), which is the deep restorative phase of sleep, which can lead to difficulties with falling asleep and staying asleep (Hagen et al., 2015; Tauman, 2012). Lower levels of leptin are associated with fragmented sleep and increased wakefulness (Hagen et al., 2015; Tauman, 2012).

Research in individuals with eating disorders (EDs) show that they suffer from disturbed sleep including highly fragmented sleep, difficulty falling asleep, fragmented sleep, early wake‐up times, reduced SWS, irregular sleeping patterns, hypersomnia and sleep‐related movement disorders (Carollo et al., 2023; Tauman, 2012). Overall, ED symptoms are more severe in patients who experience sleep disturbances than in patients without sleep disturbances (Kim et al., 2010). Sleep disturbances can contribute to poorer psychiatric treatment outcomes or response (Lombardo et al., 2015). Additionally, patients with sleep disturbances may be more susceptible to behavioural problems, such as binge‐eating and purging (Kim et al., 2010). These findings, combined with the bidirectional relationship between sleep disturbances and psychiatric disorders make the recognition and management of sleep disturbances crucial for improving treatment outcomes and preventing mental illness relapse (Abad & Guilleminault, 2005; Ivanenko & Johnson, 2008).

Anorexia nervosa (AN) is a severe psychiatric disorder characterised by abnormal eating patterns, severe self‐induced weight loss, intense fear of weight gain, and a disturbed body perception. There are two main subtypes of AN: AN‐restricting and AN‐purging. Individuals with the AN‐restricting type primarily lose weight through severe dietary restriction and/or excessive exercise, while the AN‐purging type involves regular episodes of binge eating (e.g., consuming an unusually large amount of food in a short period) followed by purging behaviours to prevent weight gain. It is estimated that about half of the patients with AN report having sleep disturbances (Kim et al., 2010; Padez‐Vieira & Afonso, 2016). Sleep disturbances in patients with AN might stem from the elevated levels of ghrelin or orexin due to prolonged starvation, and lower leptin levels due to low body fat (Misra & Klibanski, 2014; Sauchelli et al., 2016). It could also stem for comorbid psychiatric disorders like depression or anxiety, which are highly prevalent in patients with AN and are associated with impaired sleep (Alvaro et al., 2013; Lauer & Krieg, 2004). Additionally, compulsive exercise (Cinosi et al., 2011), can lead to a state of hyperarousal due to elevated cortisol levels, which disrupt sleep patterns (Klein et al., 2007). Moreover, patients with AN often suffer from nocturia, due to electrolyte imbalances, use of diuretics or elevated cortisol levels, and leg cramps due to dehydration or electrolyte imbalances (Mehler & Brown, 2015). However, currently, little is known about the specific types of sleep disturbances these patients face. Hence, this review aimed to characterise sleep (disturbances) in patients with AN by comparing various sleep metrics between patients with AN and healthy controls, as well as after weight restoration. Where possible, data were aggregated in meta‐analyses.

## 2 METHODS

This review was registered on PROSPERO (international prospective register of systematic reviews) with registration number CRD42023458807and followed the Preferred Reporting Items for Systematic Reviews and Meta‐analysis (PRISMA) guidelines. (PRISMA. Accessed 12‐09‐2020)

### 2.1 Search strategy

A systematic search was conducted in the following databases (last update on 24 May 2024): PubMed, Embase.com, Clarivate Analytics/Web of Science Core Collection and PsycINFO. The search included a combination of keywords and free text terms for (synonyms of) 'sleep', ‘insomnia’, ‘circadian rhythm’ or ‘obstructive sleep apnoea disorder’ combined with (synonyms of) 'anorexia nervosa' (see Supplementary Information S1). No restrictions were applied regarding date or language. A reference search was applied to capture any remaining eligible papers (Wohlin, 2014).

### 2.2 Inclusion/exclusion criteria

This review included papers that assess potential relationships between AN and sleep metrics (quantity and quality) using statistical analyses, as well as those describing macro/micro sleep architecture in patients with AN. Studies related to bulimia nervosa, night eating syndrome, or other types of eating disorders were excluded. Papers that do not differentiate between types of eating disorders were also excluded. Additionally, papers not published in peer‐reviewed journals, literature reviews, written in languages other than English, Dutch, or German, or based on animal studies were excluded.

### 2.3 Outcomes of interest

Upfront outcomes regarding both sleep quantity, quality and sleep architecture were established; see Table 1. Additional relevant sleep outcomes were incorporated during the data analysis if they emerged from the data and were used more than once.

**TABLE 1:** Explanation sleep parameters.

Sleep parameter	Definition/Explanation
Sleep quantity	
Sleep onset latency (SOL)	Duration between bedtime and sleep onset
Total sleep time (TST)	Total minutes of night‐time sleep (‘time in bed’ minus ‘wake after sleep onset’ minus “sleep onset latency” OR ‘sleep duration’ – ‘wake after sleep onset’)
Wake after sleep onset (WASO)	Total number of minutes spent awake after sleep onset during the sleep period at night
Sleep efficiency (SE)	Number of minutes of actual sleep (TST) during the night divided by the number of minutes in bed, multiplied by 100
Sleep quality	
Pittsburgh Sleep quality index (PSQI) (Buysse et al., 1989)	19 self‐rated questions (and 5 questions rated by the bed partner or roommate) which assesses sleep behaviour from the previous month. PSQI has 7 subcategories, which sums up to a score between 0 and 21: Subjective sleep quality, sleep latency, sleep duration, sleep efficiency, daytime dysfunctions, sleep disturbances, sleep medication). A total score ≥5 is considered poor quality.
Insomnia severity index (ISI) (Bastien et al., 2001/07)	7 self‐rated questions, using a 5‐point Likert scale, to assess the severity of sleep‐onset, sleep‐maintenance, and early awakenings, as well as the satisfaction with current sleep patterns, interference with daytime functioning, noticeability of impairment to others and level of distress. Assesses the last 2 weeks. Total scores range from 0 to 28, with scores of 0–7, 8–14, 15–21 and 22–28 deemed indicative of no clinically significant insomnia, subthreshold insomnia, moderate insomnia and severe insomnia, respectively.
Epworth sleepiness scale (ESS) (Johns, 1991)	8 self‐report items to evaluate the general sleepiness state within a day, using a 4‐point Likert type scale. Total scores range from 0 to 24, where 0–5 indicates enough sleep, 6–10 indicates slight drowsiness, 11–15 indicates average drowsiness and >15 indicates severe drowsiness.
Sleep architecture	
% In sleep stage 1	The percentage of total sleep time spent in stage 1, the lightest stage of sleep where a person can easily be awakened.
% In sleep stage 2	The percentage of total sleep time spent in stage 2, a deeper stage than stage 1, characterised by slower brain waves with occasional bursts of rapid activity.
% slow wave sleep (sleep stage 3 + 4)	The percentage of total sleep time spent in slow wave sleep (SWS), the deepest and most restorative stage of sleep.
Rapid eye movement (REM) %	The percentage of total sleep time spent in REM sleep, a stage where dreaming occurs, and the brain is highly active while the body is relaxed.
REM latency	The duration in minutes from the onset of sleep to the first occurrence of REM sleep.

### 2.4 Data extraction and quality assessment

After the initial database search and after deduplication, ASReview was used to assess the eligibility of titles and abstracts (van de Schoot et al., 2021/02). This open‐source tool employs active learning and machine learning models to aid researchers in making inclusion and exclusion decisions. It presents articles one at a time for evaluation based on initial categorisations. Researchers' decisions improve the algorithm's accuracy and prompt it to reorder the remaining articles, prioritising those deemed most relevant, thereby enhancing the review process (van de Schoot et al., 2021/02). Two researchers (PB, RB) independently screened all titles and abstracts with ASReview with the following setup: TF‐IDF as feature extraction technique, Naive Bayes as classifier, maximum as query strategy and dynamic resampling as balance strategy. The stop criterium was 150 irrelevant records. Subsequently, relevant data was collected using a structured form.

### 2.5 Synthesis of literature

For papers comparing patients with AN with either (healthy) controls or before/after weight restoration, meta‐analyses were conducted for all papers assessing the relationship between AN and different sleep metrics provided there were more than five studies to include in the analysis. Meta‐analyses were possible for TST, WASO, SOL, sleep efficiency, Stage 1 Sleep %, Stage 2 Sleep %, Slow Wave Sleep (SWS) %, REM %, and REM Latency. Studies assessing the relationship between AN and multiple sleep variables were included in all relevant meta‐analyses. Pooled mean differences were calculated using a random‐effects model. Forest plots were generated, considering the direction of expected outcomes (e.g., negative values mean patients with AN have worse sleep metrics). To evaluate the amount of heterogeneity, the I^2^ index was used, with values of 25%, 50%, and 75% suggesting low, moderate, and high between‐study variability, respectively (Higgins et al., 2003). For each meta‐analysis, the prediction interval was included, representing the anticipated mean difference in future patients (Higgins et al., 2003). Publication bias was assessed when 10 or more studies were included in the meta‐analysis, by visual inspection of funnel plots, Egger's regression test, and the trim‐fill method. The certainty of the evidence was rated using the GRADE approach. Sensitivity analysis was performed to explore the influence of study type (e.g., comparison between AN and control or AN before and after weight restoration), AN‐type, and type of sleep measurement. All (meta‐)analyses were conducted using R (A Language and Environment for, 2021). If findings from papers could not be included in a meta‐analysis, the data was analysed qualitatively.

## 3 RESULTS

**FIGURE 1:**
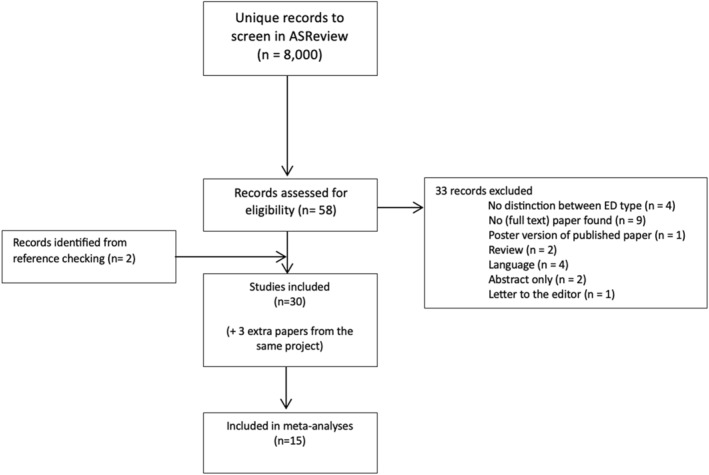
PRISMA diagram.

The search strategy initially yielded approximately 8000 unduplicated citations. Title and abstract screening identified 66 potentially eligible papers. After thorough full‐text screening, 28 papers were included in this review (see Figure 1). Reasons for excluding papers after full‐text screening included language, type of paper (e.g., reviews, letters to the editor, abstracts only), and the lack of differentiation between various EDs. Moreover, three papers were sub analyses of the same project. Additionally, two papers were added through reference checking. Of these 30 papers, 15 were included in the meta‐analysis, covering approximately 280 patients with AN. Only one paper reported sleep parameters for the AN‐P type (Delvenne et al., 1992), others exclusively included patients with AN‐R, or did not differentiate between the types. Certainty of evidence was graded very low for all meta‐analyses. Table 2 presents a detailed summary of each study.

**TABLE 2:** Summary of included papers (design, participant characteristics, sleep measurement, recruitment, findings).

Author (year)	Methodological design	Number of participants	An type	Age	Weight related measures	Disease duration	Sex (F/M)	(Used questionnaire) Comorbidities	Sleep measurement	Status patient/Recruitment	Most important findings
Asaad Abdou et al. (2018)	Cross‐sectional	14	UN	30 (6)	UN	UN	F	**Depression:** 35% minimal, 30% mild, 30% moderate.	PSG (1 night)	Both outpatients and inpatients	Patients experienced more parasomnias, daytime hypersomnolence, and insomnia compared to healthy controls. Patients experienced longer sleep latency, reduction of sleep efficiency, increased arousal index compared to healthy controls. Depression scores were positively correlated with sleep latency, stage I sleep, and REM latency, and a negative correlation to SWS.
		HC (20)									
Bat‐Pitault et al. (2021)	Retrospective cross‐sectional	111	ANR (78) ANB/P (33)	15.1 (2.1)	BMI 14.8 (2.0)	10.9 months (5.8)	F (93%)	**Anxiety** (State Trait anxiety Inventory) **Depression** (Children's depression Inventory)	PSQI, ESS, SOL	Both outpatients and inpatients	Significant higher sleep disturbances in the ANB/P group compared to ANR. Significant greater sleepiness and more eveningness chronotype in ANB/P. Poor sleepers showed significantly more severe ED symptoms and anxio‐depressive symptoms compared to good sleepers. Quality of life was significantly worse in the poor sleepers compared to the good sleepers.
Carpine et al. (2022)	Cross‐sectional	107	ANR (89) ANB/P (18)	14.8 (2.1)	BMI 14.9 (2.0)	10.4 months (5.2)	F (95%)	**Depression** (Children's depression Inventory) **Anxiety** (state Trait anxiety Inventory) **ADHD** (CONNERS 10)	PSQI, ISI	Both outpatients and inpatients	In the entire sample, the presence of excessive exercise was associated with significantly higher levels of impaired sleep.
Crisp et al. (1970) (Same project: Crisp et al. (1971), Nell et al. (1980))	Cohort (within patient)	10	UN	22 (4)	Pre‐treatment weight 39.9kg, post‐treatment weight 54.56kg	UN	F	None	EEG (4 nights)	Inpatients, 10‐14days after admission	Significant higher TST, lower SOL, lower WASO post‐treatment.
Della Marca et al. (2004)	Case‐control	AN (6)	UN	20.7 (1.9)	BMI 16.2 (0.9)	UN	F	**Depression (**Hamilton rating scale)	PSG (2 nights)	Outpatient, prior to treatment.	AN had significantly lower SE compared to HC.
		HC (6)	N/A	20.7 (1.8)	BMI 20.8 (1.9)	N/A	F				
Delvenne et al. (1992)	Case‐control	AN (11)	ANR (5) ANB/P (6)	18 (3)	%IBW 71 (11)	UN	F	1 female with Major depression disorder, others no comorbidity	EEG (2–3 nights)	Inpatients, approximately 1 week after admission.	In comparison with controls, patients with AN showed lower sleep efficiency, higher length of awakenings and less REM sleep. No significant correlation between sleep variables and BMI were found. Higher length of awakenings, an increase in stage 3 sleep, and a decrease in REM sleep was found in patients with ANR compared to patients with ANB/P.
		HC (11)	N/A	18 (3)	%IBW 104 (15)	UN	F				
El Ghoch et al. (2016)	Case‐control including within patient comparisons	AN (50)	ANR	24.6 (8.9)	BMI 24.6 (8.9)	8.9 years (8.4)	F	None	Actigraphy (4 nights)	Inpatients	Pre‐treatment patients with AN showed significantly lower TST, shorter SOL compared to controls, but no significant difference for SE and WASO. Pre‐treatment sleep was associated negatively associated with age and duration of illness, and positively with pre‐treatment BMI Post‐treatment patients with AN showed a significant improvement of TST compared to pre‐treatment, but no significant difference for SOL, SE or WASO.
		HC (25)	N/A	26.3 (10.6)	BMI 21.5 (1.8)	N/A	F				
Kleppe et al. (2023)	Case‐control	AN (20)	UN	19.5 (8.0)	BMI 16.7 (2.8)	8.7 years (range 1–33)	F	**Depression** (Beck depression Inventory‐II)	Actigraphy (1w)	Outpatients	No differences were found between patients with AN and HC regarding average sleep onset, TST, SE, awakenings. Patients with AN had significant shorter WASO, but longer duration of mid‐sleep awakenings lasting >5 min No correlation was found between BMI and sleep variables. A correlation was found between sleep efficiency and depression score.
		HC (23)	N/A	19.0 (7.0)	BMI 22.9 (3.5)		F				
Lacey et al. (1976) (Same project: (Lacey et al. (1975))	Cohort (within patient)	10	ANR	UN	Pre‐treatment weight 37.6kg (5.1), post‐treatment weight 52.0kg (4.6)	UN	F (90%)		EEG (2x3 nights)	Inpatients	After reaching target weight, a significant increase in TST, a significant decrease of wakefulness (hours spent awake per hour), and an increase of REM sleep was found.
Langlet et al. (2021)	Cross‐sectional	67	UN	17.8 (6.9)	BMI 15.5 (1.9)	3.3 (5.3)	F (93%)	None	Actigraphy (1w)	Inpatients, within 1 week after admission.	31% of the patients met sleep recommendations.
Latzer et al. (2001)	Case‐control	AN (21)	ANR (15)ANB/P (6)	18.7 (3.5)	BMI 16.8 (1.3)	UN	F	4 patients with mental comorbidities	Actigraphy (1w) MSQ	Outpatients, prior to treatment.	Self‐reported sleep disturbances as significantly higher in patients with AN compared to controls. No significant differences in objective sleep metrics were observed.
		HC (16)	N/A	19.4 (3.9)	BMI 20.0 (1.3)	N/A	F				
Lauer et al. (1989)	Case‐control	AN (20)	UN	21 (3)	%IBW 70.3 (6.3)	UN	F (95%)	**Depression** (inpatient Multidimensional psychiatric scale): 9 with current and/or lifetime diagnosis of major depression.	PSG (3 nights)	Inpatients, on average 11dayays after admission	No statistically significant group effects were found for sleep metrics or sleep architecture. There was no significant correlation between body weight (% IBW) and sleep parameters.
		HC (10)	N/A	23 (3)	%IBW 96.0 (5.9)	N/A	F (90%)		PSG (2 nights)		
Lauer et al. (1989)	Cross‐sectional	10	UN	20.6 (2.8)	%IBW 70.7 (5.9)	3.3 years (2.6)	F	None	PSG (2 nights)	Outpatients, under treatment	x
Lauer et al. (1992)	Case‐control including within patient comparisons	AN (10)	ANR (3) ANB/P (7)	20.0 (3.0)	%IBW 70.2 (7.4)	3.5 years (2.0)	F	None	PSG	Unknown	Before weight gain, the only significant difference between patients and controls was an increase in SWS in patients. Compared to pre‐treatment, stage 3 sleep was significantly reduced after treatment, for the benefit of an increase of stage 4 sleep. The duration of the first REM period was significantly prolonged post‐treatment. There was no correlation between %IBM and the degree of sleep changes.
		HC (10)	N/A	23.1 (3.1)	%IBW 96.0 (5.9)	N/A	F				
Levy et al. (1987) (Same project: (Levy et al. (1988))	Case‐control	AN (6)	ANR ANB/P	24.8 (6.8)	%IBW 70.5 (8.6)	UN	F	**Depression** (Beck Depression Inventory, Hamilton rating scale for depression): 1 minor depression, 2 major depression	PSG (2 nights)	Inpatients	Slow wave sleep was significantly lower in patients with ANR compared to controls, while there was no difference in SWV for ANB/P and controls. There was no difference between AN patients and REM latency or total minutes in REM sleep. Patients with AN had significantly less TST and sleep efficiency, and longer WASO than controls. They spent a greater percentage of sleep time in stage 1.
		HC (10)		23.2 (2.4)	%IBW 103.0 (7.6)	N/A	F				
Lim et al. (2023)	Cross‐sectional	33	UN	23.4 (2.6)	BMI 19.2 (1.0)	UN	M	**Depression/Anxiety** (depression anxiety stress scale)	Actigraphy	Outpatients	A positive relationship was found between sleep efficiency and BMI (but not with TST), while no significant relationship was found between ED severity and TST or sleep efficiency.
Lindberg et al. (2003)	Case‐control including within patient comparisons	AN (11)	ANR	19.7 (1.1)	13.3 (0.3)	2.2 years (0.6)	F	**Depression (**Montgomery‐Asberg depression rating scale).	PSG (2 nights), and actigraphy.	Inpatients, within 1 week after admission.	The actigraphic analysis did not reveal any significant differences between patients with AN and healthy controls. Polysomnography showed that patients with AN had significantly less TST, SE, and SWS than healthy controls. Additionally, more stage 1 sleep. SWS significantly increased after weight gain, while both stage 1 and stage 2 decreased.
		HC (11)		20.9 (0.8)	21.4 (0.5)	N/A	F				
Malcolm (2022)	Case‐control	AN (96)	UN	27.9 (7.1)	BMI 20.1 (3.5)	UN	F (78%)	**Depression/Anxiety** (depression anxiety stress scale)	PSQI	Outpatients	Patients with AN reported significantly poorer sleep quality compared to controls, and higher anxiety and depression scores. Poorer sleep was significantly correlated with increased severity of ED symptoms, higher depression/anxiety scores and lower BMI.
		HC (246)	N/A	30.7 (9.83)	BMI 25.1 (4.8)	N/A					
Martínez‐Sánchez et al. (2020)	Cohort	14	ANR	14.3 (1.6)	BMI 17.9 (2.6)	UN	F	No patients with mental comorbidities.	Actigraphy (9 days)	Outpatients in a 10 weeks treatment programme	After treatment, patients had significant weight gain. With regard to sleep, there was a significant increase in sleep latency and a decrease in sleep efficiency. There was no significant difference regarding TST and awakenings.
Martínez‐Sánchez et al. (2020)	Quasi experimental study	12	ANR, ANB/P, Atypical AN	14.6 (1.7)	BMI 19.6 (2.2)	UN	F	No patients with mental comorbidities.	Actigraphy (9 days)	Participating in a Pilates programme, outpatients	x
Nobili et al. (2004)	Case‐control	AN (20)	ANR	13.9 (2.0)	IBW% 69% (13)	14.0 months (10)	F	No patients with mental comorbidities.	EEG (2 nights)	Inpatients, during first week after admission.	Patients with AN showed an increased number of awakenings, a higher arousal index, more WASO, and decreased SWS compared to controls. No differences were found for stage 1 and stage 2 sleep.
		HC (12)	N/A	14.0 (2.0)	IBW% 106 (7.6)	N/A	F				
Nobili et al. (1999)	Case‐control	AN (10)	ANR	14.0 (2.0)	IBW% 67.0 (11.0)	UN	F	No major depression	EEG (2 nights)	Inpatients, during first week after admission.	Patients with showed an increased number of awakenings, increased WASO, and a reduction in sleep efficiency compared to healthy controls.
		HC (10)	N/A	14.0 (2.0)	IBW% 106.0 (7.6)	N/A	F				
Pauls et al. (1991)	Case report	1	ANB/P	28years	BMI 14.9 (2.0)	2years	M		EEG	Inpatients	EEG showed reduced sleep time, lower sleep efficiency and reduced REM sleep. Basal insulin and triiodothyronine concentrations were reduced, cortisol and growth hormone levels were elevated.
Pieters et al. (2004)	Cohort (within patient)	58	ANR	19.4 (4.4)	BMI 14.6 (1.6)	3.5 years (2.9)	UN	**Depression/anxiety** (Hamilton depression and anxiety scale)	PSG (2 nights) PSQI	Inpatient, shortly after admission and second measurement when reaching a BMI of 19 (mean interval 136 days (35.7).	Sleep quality was significant higher at T1 compared to T2. No significant changes in polysomnography metrics between T1 and T2 (TST, sleep efficiency, awakenings, sleep latency, REM latency, SWS, light sleep). Depression and anxiety significantly improved. Percentage of underweight at admission and the amount of SWS were predictions for the length of time required for weight restoration.
Romigi et al. (2022)	Case‐control	AN (34)	ANR	23.3 (6.5)	BMI 16.5 [15.3–17.6)	UN	F	No patients with mental comorbidities. **Depression** (Beck depression Inventory)	PSQI ESS	Outpatients	Sleep quality was significantly lower in patients with AN compared to controls. Patients with AN did not experience significant more daytime drowsiness. Sleep quality was significantly correlated with quality of life in patients with AN.
		HC (24)	N/A	23.3 (6.5)	BMI 21.0 [19.8–23.3]	N/A	F				
Rossi et al. (2023)	Cross‐sectional	AN (101)	ANR (82)ANB/P (19)	14.7 (2.2)	BMI 14.6 (1.6)	12.9 years (8.1)	F (98%)	**Depression** (Children's depression Inventory) **Anxiety** (state Trait anxiety Inventory)	PSQI ISI	Outpatients	Adolescents self‐reported significantly more sleep difficulties and lower rates of positive emotions during COVID lockdown, compared to those evaluated before it. Furthermore, present findings overall suggest that as the lockdown progresses, adolescents with AN show an increased deterioration of sleep quality, as indicated by greater insomnia severity levels.
Sauchelli et al. (2016)	Case‐control	AN (48)	UN	27.2 (8.7)	BMI <18.5		F	Symptom Checklist revised	PSQI	Inpatients, before start of treatment	Patients with presented more sleep disturbances and poorer overall sleep quality than did the healthy controls. Orexin‐A concentrations were associated with greater sleep disturbances and poorer overall sleep. Both elevated orexin‐A concentrations and inadequate sleep predicted poorer treatment outcome.
		HC (98)	N/A	27.5 (7.9)	BMI 18.5–24.9		F				
Tanahashi et al. (2017)	Cross‐sectional	20	ANR (8)ANB/P (12)	28.6 (13.1)	BMI 13.3 (2.2)	7.2 years (8.9)	F	**Depression** (Centre of Epidemiologic studies depression scale)	PSQI	Inpatients	Circadian rhythm disruptions and abnormal sleep durations were significantly greater in patients with ANB/P than in patients with ANR. Sleep quality was significantly correlated with a diagnosis of ANB/P, vomiting, and duration of illness.
Walsh et al. (1985)	Case‐control	AN (8)	ANR (3)ANB/P (5)	26.9 (6.9)	%IBW 64.4 (6.7)	5.5 years (4.0)	F	4 with major depression	EEG (2 nights)	Inpatients	Patients with AN spent less time in stage 2 compared to healthy controls. They have significantly lower TST. Significant negative correlations were found between %IBW and percentage of sleep in stage 1, and REM density. There was a positive correlation between %IBW and REM cycle length. Patients with both AN and Major depression had shorter REM latencies than patients without major depression; while there were no other differences between depressed and nondepressed patients.
		HC (14)	N/A	26.6 (5.0)	%IBW 95.0 (8.9)	N/A	F				

Abbreviations: %IBW, percentage of ideal body weight; AN, anorexia nervosa; ANR, anorexia nervosa restrictive type; ANB/P, anorexia nervosa Bulimic/Purging type; BMI, body Mass index; EEG, electroencephalogram; ESS, Epworth sleepiness scale; F, female; HC, healthy control; ISI, insomnia severity index; M, male; N/A, not applicable; PSG, polysomnography; PSQI, Pittsburgh Sleep quality index; UN, Unknown.

### 3.1 Sleep quantity

The average mean difference for TST was −32.1 min ([95%‐CI: −50.9, −13.4], *I* ^2^ = 86%, *p* = 0.003, Figure S2.1), indicating lower TST in patients with AN. The mean difference was larger for studies comparing AN with HC (−38.7 [95%‐CI: −59.5, −18.0]) compared to studies comparing AN before and after weight restoration (−3.7 [95%‐CI: −21.9, 14.5]). No publication bias was observed.

The meta‐analysis for SOL did not show any significant differences between patients with AN and HC or after weight restoration (Figure S2.2). Sensitivity analyses did not reveal any differences. The meta‐analysis for WASO showed an average mean difference of 18.9 ([95%‐CI: −2.4, 40.2], *I* ^2^ = 83%, *p* = 0.074 Figure S2.3), indicating longer WASO in patients with AN. Studies using PSG showed higher mean differences compared to studies using actigraphy (27.5 [95%‐CI: 3.0, 52.0] vs. −6.2 [95%‐CI: −71.0, 58.6]).

The meta‐analysis for sleep efficiency showed an average mean difference of −4.4% ([95%‐CI: −7.9, −0.9], *I* ^2^ = 79%, *p* = 0.018, Figure S2.4), suggesting lower sleep efficiency in patients with AN. Studies using PSG showed higher mean differences compared to studies using actigraphy (27.5 [95%‐CI: 3.0, 52.0] vs. −6.2 [‐71.0; 58.6]). The mean difference was larger for studies comparing AN with HC (−5.6 [95%‐CI: −9.3, −1.9]) compared to studies comparing AN before and after weight restoration (1.2 [95%‐CI: −15.3, 17.7]). Additionally, the mean difference was larger for studies using polysomnography (PSG) (i.e., the reference standard for measuring sleep) compared to actigraphy (i.e., a wrist‐worn device that estimates sleep based on body movements) (−6.1 [95%‐CI: −9.9, −2.4] vs. 1.0 [95%‐CI: −2.5, 4.5], respectively). No publication bias was observed.

### 3.2 Sleep quality, insomnia, and sleepiness

There were insufficient papers comparing patients with AN with HC to perform a meta‐analysis for sleep quality. All studies that assessed sleep quality using the PSQI reported a mean or median score greater than 5, suggesting poor sleep quality in patients with AN (Bat‐Pitault et al., 2021; Carpine et al., 2022; Malcolm et al., 2022; Pieters et al., 2004; Romigi et al., 2022; Rossi, Silva, Charvin, Da Fonseca, & Bat‐Pitault, 2023; Sauchelli et al., 2016; Tanahashi, Kawai, Tatsushima, & et al., 2017). Three studies compared PSQI scores with HC and found significantly higher scores in patients with AN (Malcolm et al., 2022; Romigi et al., 2022; Sauchelli et al., 2016). Additionally, one study compared sleep quality before and after weight restoration, which was significantly improved. Two studies showed a significantly higher PSQI score for patients with AN‐P type compared to AN‐R (*p* < 0.05) (Bat‐Pitault et al., 2021), while another study showed a significantly higher PSQI score for patients with the presence of ‘problematic physical activity’ (PPA) compared to patients without PPA (*p* = 0.008) (Carpine et al., 2022).

Few studies have investigated the association between daytime sleepiness and AN, but they did not find an association (Bat‐Pitault et al., 2021; Romigi et al., 2022; Wilcox et al., 2024). No significant difference in ESS was found between patients with AN and HC (Romigi et al., 2022). Additionally, one study assessed ISI scores, which showed mean ISI score 7.7 (5.7), suggesting the absence of moderate insomnia (Rossi, Silva, Charvin, Fonseca, & Bat‐Pitault, 2023Rossi, Silva, Charvin, Fonseca, & Bat‐Pitault, 2023). Finally, one study showed that patients with comorbid sleep disturbance showed clinically severe symptoms and more disturbing behavioural symptoms compared to patients with AN that did not have sleep disturbances (Kim et al., 2010).

### 3.3 Sleep architecture

The meta‐analysis (7 out of 8 papers included) for Stage 1 Sleep % showed an average mean difference of 2.4% ([95%‐CI: 0.05, 4.7], *I* ^2^ = 93%, *p* = 0.0463, Figure S2.5). The meta‐analysis (9 out of 10 papers included) for REM% showed an average mean difference of −2.1% ([95%‐CI: −4.2, −0.02], *I* ^2^ = 61%, *p* = 0.0483, Figure S2.6). The mean difference for REM% was larger for studies comparing AN with HC (−2.3 [95%‐CI: −4.9, 0.27]) compared to studies comparing AN before and after weight restoration (−1.1 [95%‐CI: −5.7, 3.5]). There were no statistical differences for Stage 2 sleep %, SWS % and REM Latency (see Figures S2.7–9).

## 4 DISCUSSION

This review aimed to characterise sleep (disturbances) in AN. The findings suggest that patients with AN generally experience significantly lower TST, higher WASO, and lower sleep efficiency compared to HC. We did not find significant changes in TST and sleep efficiency before and after weight restoration in patients with AN, indicating that weight restoration alone may not be sufficient to improve sleep. Anxiety triggered by the increase in weight and the pressure of being monitored by healthcare professionals could be one of the explanations for the lack of sleep improvement (Kim et al., 2010), or the existence of other comorbid psychiatric disorders. Additionally, weight restoration does not mean that the eating disorder is in remission, so there might be other psychological, environmental, or neurobiological factors that could explain the lack of improvement in sleep after weight restoration. For example, research indicates that patients with AN experience significant thalamocortical and cortical changes (which is important for slow wave activity), including reduced cortical thickness in frontal and cingulate regions and white matter abnormalities (Nobili et al., 2004b). Recovery of these brain structures is possible with weight restoration, though the extent and duration of recovery varies (Lázaro et al., 2013; Nickel et al., 2018).

Our review shows inconsistent findings regarding sleep architecture. The meta‐analysis showed a significant increase in Stage 1 sleep and a decrease in REM sleep. Although our meta‐analysis did not show a significant difference in SWS, most studies investigating SWS did find differences between patients with AN and HC (Asaad Abdou et al., 2018; Levy et al., 1988; Lindberg et al., 2003; Nobili et al., 2004b). This might be explained by the small sample size and heterogeneity in patients with AN, since most studies did not differentiate between AN‐R and AN‐P type. Stage 1 is a light sleep stage where individuals can be easily awakened. An increase in stage 1 sleep suggests more fragmented and less restorative sleep. A decrease in SWS can lead to impaired physical recovery and a weakened immune system (Lange et al., 2010), possibly making it harder for patients with AN to recover from malnutrition and other physical complications associated with the disorder. Additionally, a decrease in REM sleep can negatively affect cognitive performance, emotional regulation and overall mental health (Kusztor et al., 2019). This could contribute to the cognitive distortions and emotional dysregulation often seen in patients with AN. The changes in sleep architecture in AN might be explained through malnutrition. Malnutrition leads to physical and psychological stress, which can increase arousal and difficulty in achieving SWS (Griffiths et al., 2023). Additionally, malnutrition might impair the restorative processes, potentially because the body lacks the necessary nutrients for SWS (Gratwicke et al., 2021). Moreover, hormonal imbalances caused by malnutrition, such as reduced levels of growth hormone, which peaks during SWS, might further reduce the time spent in this sleep stage (Zaffanello et al., 2024). Additionally, malnutrition also affects neurotransmitter levels (e.g., serotonin and dopamine), that regulate sleep cycles, particularly REM sleep (Fraigne et al., 2023).

Our meta‐analysis showed that there is a significant difference between patients with AN and HC regarding TST, WASO and sleep efficiency. However, there is no difference for these sleep parameters before and after weight restoration. It could be that the malnutrition in patients with AN causes lasting changes in the neurotransmitter system, even after weight restoration. Research has shown that while many brain changes due to starvation are reversible, some alterations in neurotransmitter systems, particularly those involving serotonin and dopamine, may persist (Marzola et al., 2013; Mysliwiec, 2020). Alternatively, sleep habits developed during the period of malnutrition may persists, leading to continued sleep issues (Mutti et al., 2023). Another explanation might be that recovery from disease takes longer than is anticipated in the studies, which would in turn result in a lack of difference between the measurements.

Future research could focus on several key areas to better understand and address the relationship between AN and sleep disturbances, especially given the very low quality of evidence for all meta‐analyses. Observational longitudinal studies are needed to track sleep patterns after weight restoration, while exploring the role of other comorbidity and psychological factors in exacerbating sleep impairment. Detailed examinations of sleep architecture and the impact of malnutrition and hormonal imbalances on sleep stages are essential. These studies should consider different subpopulations of patients with AN, various settings (e.g., home vs. hospital), and incorporate both subjective and objective sleep measurements. Lastly, treatment approaches that combine nutritional rehabilitation, psychological support, and sleep‐specific therapies could be developed and tested to assess their merits in patient outcomes.

This review has significant clinical implications for understanding the relationship between sleep and AN. First, the evidence suggests that poor sleepers with AN tend to exhibit more severe ED symptoms and higher levels of anxiety and depressive symptoms compared to good sleepers (Bat‐Pitault et al., 2021; Malcolm et al., 2022). This indicates that sleep quality may be a critical factor in the psychological and emotional well‐being of patients with AN. However, it is important to note that some studies have not found a clear association between sleep disturbances and eating disorder characteristics or general psychopathology (El Ghoch et al., 2016; Lim et al., 2023; Nobili et al., 1999). This suggests that TST may be more directly impacted by the severity of malnutrition and the duration of illness, rather than psychopathology alone. Clinicians could, therefore, consider the potential influence of malnutrition and chronicity on sleep when evaluating patients with AN. However, our meta‐analsyis showed that weight restoration alone does not significantly improve sleep in AN patient, suggesting the need for direct interventions for those patients with AN suffering from sleep impairment. Current guidelines mention the importance of proper sleep, but lack any detail. (Standaarden) Regular sleep assessments could help in identifying specific sleep disturbances. Nutritional rehabilitation could include micronutrients like magnesium and zinc to support sleep regulation (MOON, 2011; Zhang et al., 2022). Long‐term follow‐up is also important as sleep problems can persist post‐recovery. Addressing sleep habits by establishing consistent sleep routines and promoting balanced physical activity and screen time is vital. Additionally, one study found that the percentage of underweight at admission and the amount of SWS were predictive of the time required for weight restoration (Pieters et al., 2004). This suggests that objective sleep metrics, such as SWS, may serve as clinical markers for recovery progress, offering valuable insights into treatment planning. Moreover, sleep quality was found to be significantly related to patients' overall quality of life (Romigi et al., 2022). Given the impact of sleep on quality of life, addressing sleep disturbances could improve both mental and physical health outcomes in patients with AN.

The strength of this review lies in its systematic search, the inclusion of both objective and subjective sleep metrics, and the meta‐analyses conducted. However, it was limited by the heterogeneity of the studies. Additionally, the language was restricted to English, Dutch, and German. Furthermore, we included an overview of differences in sleep architecture, indicating the frequency with which certain differences have been reported in the literature. However, the frequency of reporting does not necessarily reflect the magnitude of the differences found. Lastly, we could not control for anxio‐depressive symptoms in our meta‐analyses.

## 5 CONCLUSION

Sleep in patients with AN is impaired, characterised by lower TST and sleep efficiency, higher WASO, more time spent in stage 1 sleep, and less time in REM sleep. Weight restoration alone may not be sufficient to improve sleep in AN patients. While further research is needed to investigate the impact of AN on sleep and to develop effective ways to improve sleep, we suggest that incorporating a focus on sleep into current treatment practices is already advisable.

## CONFLICT OF INTEREST STATEMENT

The authors declare no conflict of interest

## Supporting information

Supporting Information S1

## Data Availability

Data sharing is not applicable to this article as no new data were created or analysed in this study.
