# Supplementary material for: Sleep disturbances in anorexia nervosa
Source: Eur Eat Disord Rev. 2024 Oct 23;33(2):318–42. doi: 10.1002/erv.3148 (PMC11786938; doi:10.1002/erv.3148)
Supplement: Supplementary file 1 — Supporting Information S1 [file ERV-33-318-s001.pdf]

Supplemental information
Sleep disturbances in Anorexia Nervosa

Table of Contents

S1. Search Strategy **3**

Combine MeSH and TIAB *3*

Embase *3*

PsychInfo *3*

S2. Meta-analyses **5**

S1. Search Strategy

Combine MeSH and TIAB

**TERM**	**MeSH + TIAB**	**Comments**
Sleep (quality)	"Sleep"[Mesh] OR<br>**"Sleep"[Mesh] OR "Sleeping Habit*"[tiab] OR (("sleep*"[tiab]) AND (disturb*[tiab] OR poor*[tiab] OR qualit*[tiab] OR inadequate*[tiab] OR quantit*[tiab] OR duration*[tiab] OR "time"[tiab] OR "need"[tiab] OR "insufficien*"[tiab] OR "requirement*"[tiab] OR "efficienc*"[tiab] OR "latenc*"[tiab] OR "onset*"[tiab] OR "awakening*"[tiab] OR "disrupt*"[tiab] OR "nighttime*"[tiab] OR "interruption*"[tiab] OR "characteristic*"[tiab] OR "deprivation*"[tiab]) OR "insomnia"[tiab] OR "circadian rhythm"[tiab] OR "hypersomnia"[tiab] OR "obstructive sleep apnea"[tiab])**	
Anorexia	**"Feeding and Eating Disorders"[MeSH Terms] OR ("anorexia" [tiab] AND "nervosa"[tiab]) OR "anorexia nervosa" [tiab] OR ("eating" [tiab] AND "disorder"[tiab]) OR "eating disorder*"[tiab] OR ("purging" [tiab] AND "disorder"[tiab]) OR "purging disorder" [tiab] OR "night eating syndrome"[tiab] OR anorexi*[tiab] OR bulimi*[tiab] OR ((bing*[tiab] OR compulsive*[tiab]) AND (eat*[tiab] OR vomit*[tiab])) OR ((self induc*[tiab] OR selfinduc*[tiab]) AND vomit*[tiab])**<br><br>**"Anorexia Nervosa"[MeSH Terms] OR("anorexia" [tiab] AND "nervosa"[tiab]) OR "anorexia nervosa" [tiab] OR anorexi*[tiab]**	

Embase

**TERM**	**MeSH + TIAB**	**Comments**
Sleep (quality)	'night sleep'/exp OR 'night sleep' OR 'sleep pattern'/exp OR 'sleep pattern' OR 'sleep quality'/exp OR 'sleep quality' OR 'sleep time'/exp OR 'sleep time' OR (sleep* NEAR/3 (disturb* OR habit* OR poor* OR qualit* OR inadequate* OR quantit* OR duration* OR time OR need OR insufficien* OR requirement* OR efficienc* OR latenc* OR onset* OR awakening* OR disrupt* OR nighttime* OR interruption* OR characteristic* OR deprivation*)) OR 'insomnia' OR 'circadian rhythm'/exp OR 'circadian rhythm':ti,ab,kw	
Anorexia	**'anorexia'**/exp OR (**anorexi*** NEAR/3 **nervosa**):ti,ab,kw	

PsychInfo

Sleep (quality)	DE ("Sleep Onset" OR "Sleep Deprivation" OR "Sleep Wake Cycle") OR TI (((sleep* N3 (habit* OR disturb* OR poor* OR qualit* OR inadequate* OR quantit* OR duration* OR time OR need OR insufficien* OR requirement* OR efficienc* OR latenc* OR onset* OR awakening* OR disrupt* OR nighttime* OR interruption* OR characteristic* OR deprivation*)) **OR "insomnia" OR "circadian rhythm"OR "hypersomnia"OR "obstructive sleep apnea"**)) OR	

	AB (((sleep* N3 (habit* OR disturb* OR poor* OR qualit* OR inadequate* OR quantit* OR duration* OR time OR need OR insufficien* OR requirement* OR efficienc* OR latenc* OR onset* OR awakening* OR disrupt* OR nighttime* OR interruption* OR characteristic* OR deprivation*)) **OR "insomnia" OR "circadian rhythm"OR "hypersomnia"OR "obstructive sleep apnea"**))<br>OR<br>KW (((sleep* N3 (habit* OR disturb* OR poor* OR qualit* OR inadequate* OR quantit* OR duration* OR time OR need OR insufficien* OR requirement* OR efficienc* OR latenc* OR onset* OR awakening* OR disrupt* OR nighttime* OR interruption* OR characteristic* OR deprivation*)) **OR "insomnia" OR "circadian rhythm"OR "hypersomnia"OR "obstructive sleep apnea"**))	
Anorexia	DE anorexia nervosa OR TI ((anorexia nervosa OR anorexi*)) OR AB ((anorexia nervosa OR anorexi*)) OR KW ((anorexia nervosa OR anorexi*))	

S2. Meta-analyses

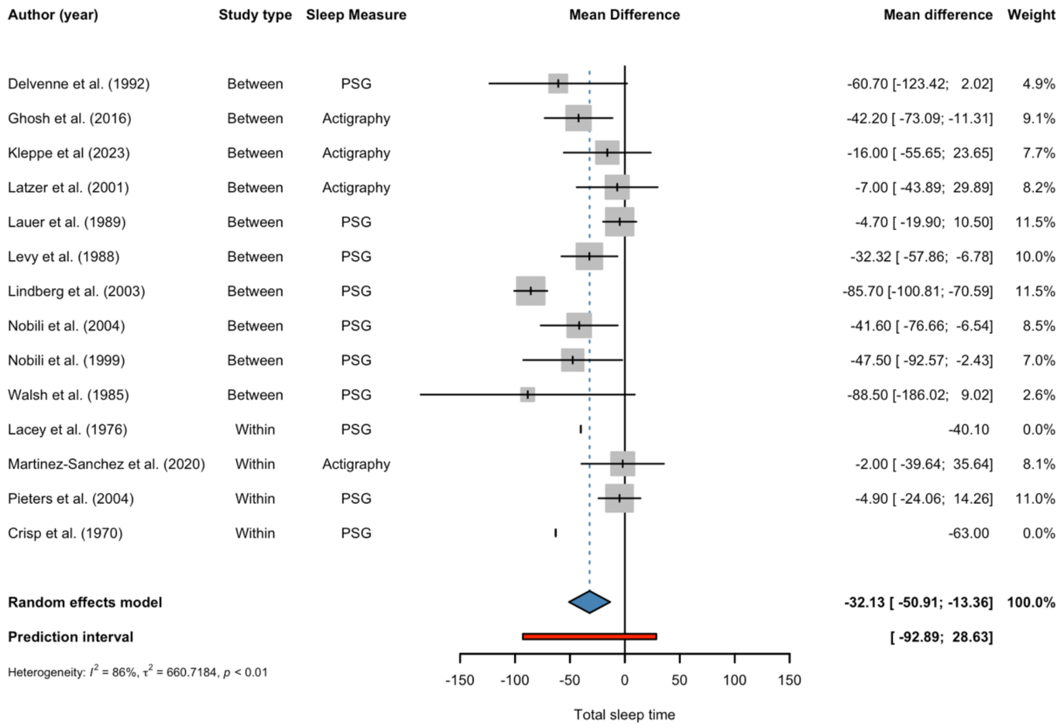

Figure 1 Meta-analysis Total sleep time

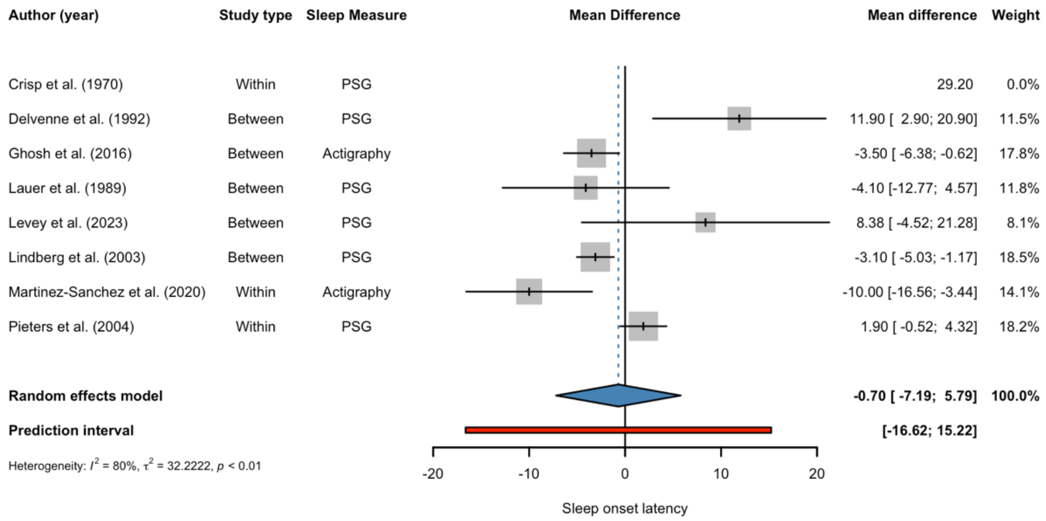

Figure 2 Meta-analysis Sleep onset latency

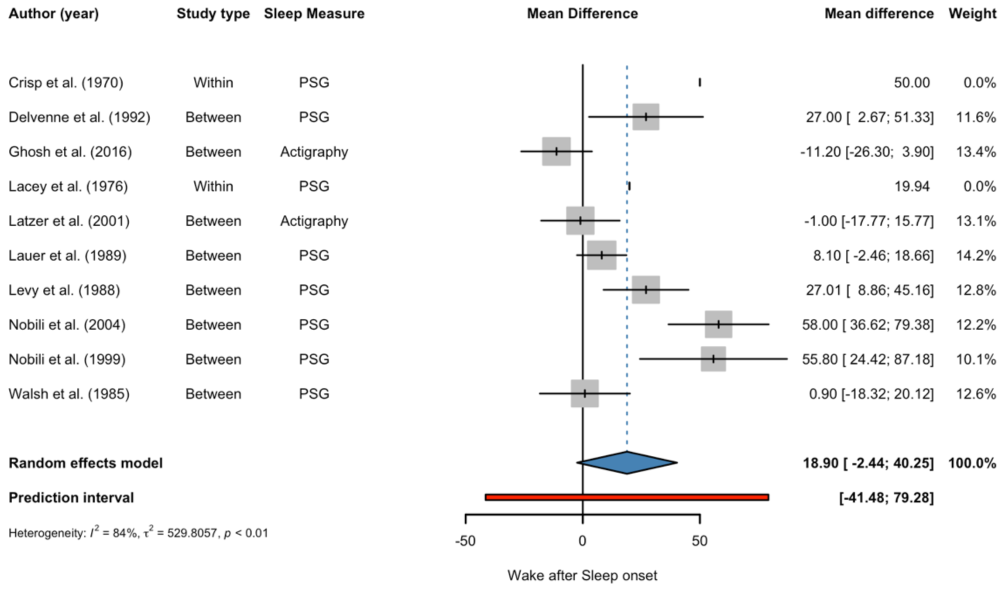

Figure 3 Meta-analysis Wake after sleep onset

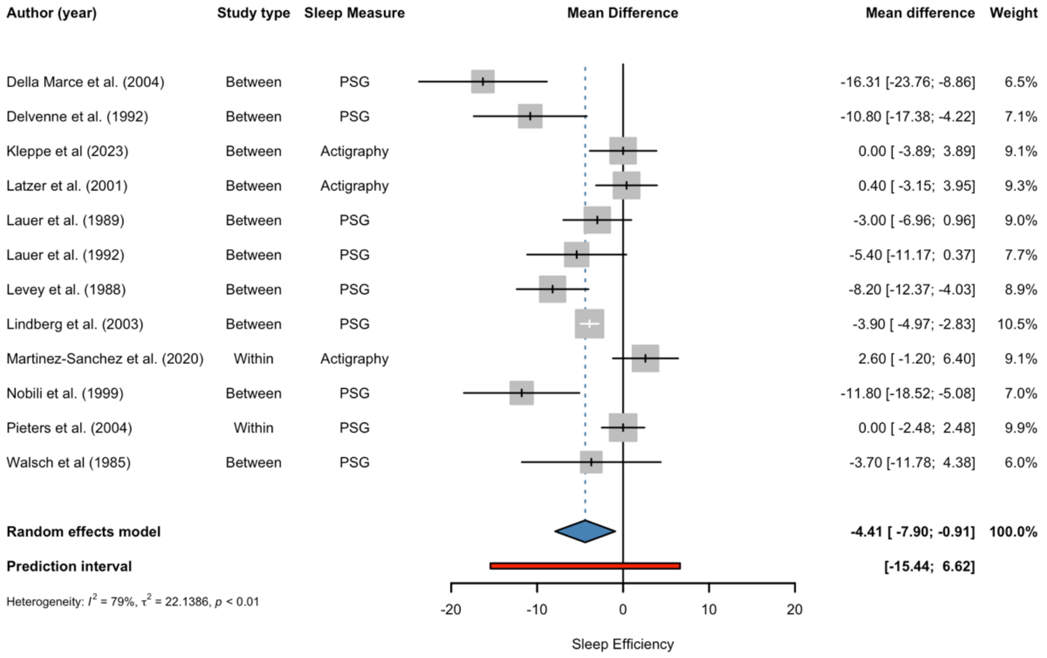

Figure 4 Meta-analysis sleep efficiency

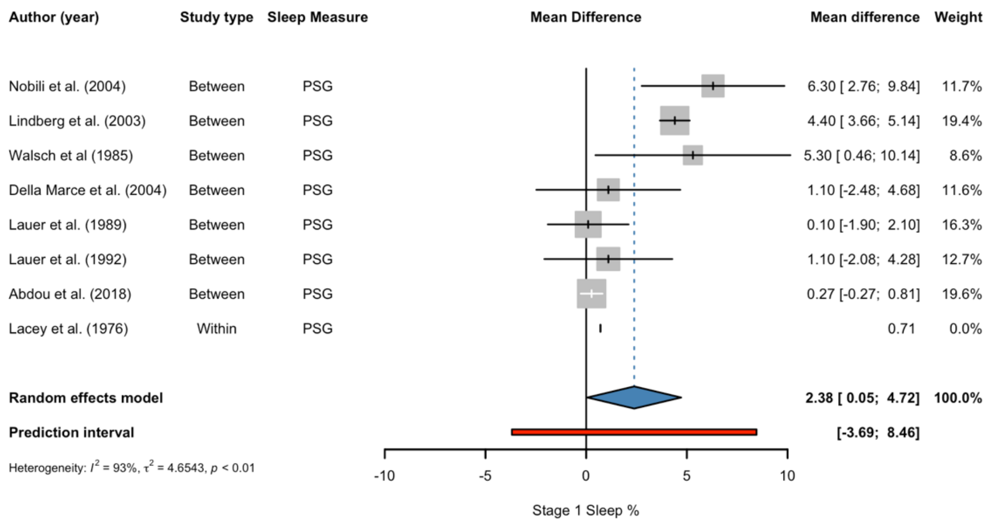

Figure 5 Meta-analysis Sleep stage 1

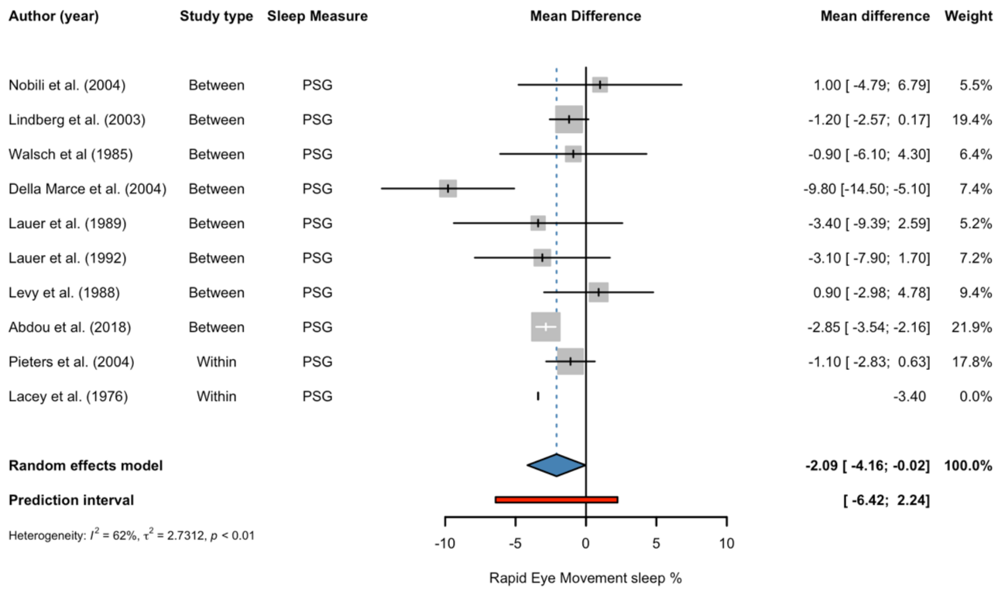

Figure 6 Meta-analysis Rapid eye movement sleep

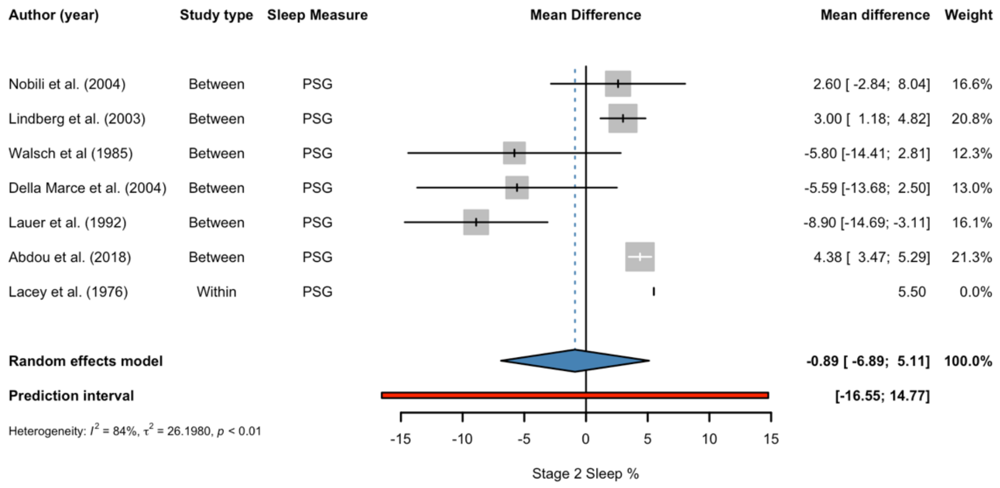

Figure 7 Meta-analysis Sleep stage 2

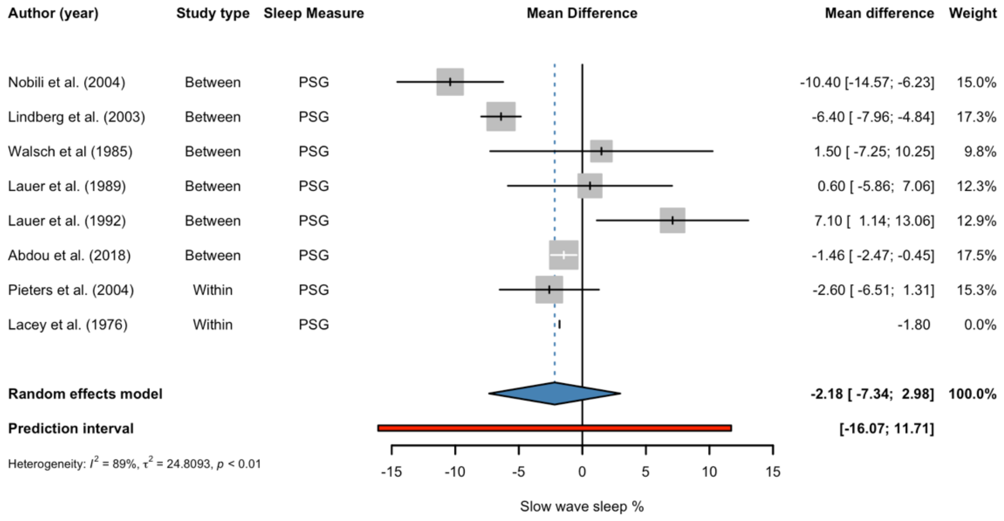

Figure 8 Meta-analysis Slow wave sleep

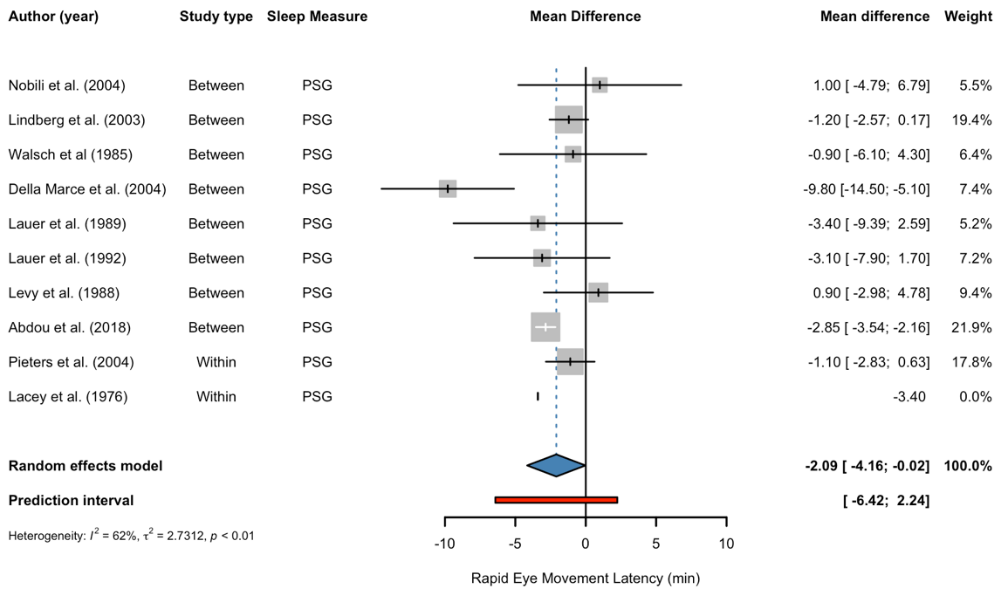

Figure 9 Meta-analysis Rapid eye movement latency
